# Reduce, reinforce, and replenish: safeguarding the early-life microbiota to reduce intergenerational health disparities

**DOI:** 10.3389/fpubh.2024.1455503

**Published:** 2024-10-23

**Authors:** Darlene L. Y. Dai, Charisse Petersen, Stuart E. Turvey

**Affiliations:** Department of Pediatrics, BC Children’s Hospital, University of British Columbia, Vancouver, BC, Canada

**Keywords:** socioeconomic status, health disparity, SES inequity, early-life exposures, intergenerational factors, microbiota

## Abstract

Socioeconomic (SE) disparity and health inequity are closely intertwined and associated with cross-generational increases in the rates of multiple chronic non-communicable diseases (NCDs) in North America and beyond. Coinciding with this social trend is an observed loss of biodiversity within the community of colonizing microbes that live in and on our bodies. Researchers have rightfully pointed to the microbiota as a key modifiable factor with the potential to ease existing health inequities. Although a number of studies have connected the adult microbiome to socioeconomic determinants and health outcomes, few studies have investigated the role of the infant microbiome in perpetuating these outcomes across generations. It is an essential and important question as the infant microbiota is highly sensitive to external forces, and observed shifts during this critical window often portend long-term outcomes of health and disease. While this is often studied in the context of direct modulators, such as delivery mode, family size, antibiotic exposure, and breastfeeding, many of these factors are tied to underlying socioeconomic and/or cross-generational factors. Exploring cross-generational socioeconomic and health inequities through the lens of the infant microbiome may provide valuable avenues to break these intergenerational cycles. In this review, we will focus on the impact of social inequality in infant microbiome development and discuss the benefits of prioritizing and restoring early-life microbiota maturation for reducing intergenerational health disparities.

## Introduction

Socioeconomic disparities are correlated with significant health inequities, as a lower socioeconomic status (SES) increases the likelihood of NCDs, such as chronic respiratory diseases, cardiometabolic diseases, oral diseases, and mental illnesses (1–5). Although researchers have identified environmental mediators, the mechanisms through which SE inequity becomes physiologically embedded remain unclear, especially regarding cross-generational health disparities that can be observed at a very young age (6–8). The observation that children from lower SES families are more likely to experience risk factors for NCDs such as obesity, cardiovascular risk, and behavioral difficulties, is alarming, as many of these diseases are associated with comorbidities that can significantly alter their lifelong health trajectories (1, 8). Thus, research into the biological underpinnings linking lower SES to greater disease risk, with a specific focus on perinatal and early-life risk factors, is increasingly needed.

Our microbiota is a significant factor bridging environmental exposures with host physiology or the microbial community that lives in/on our bodies. In this review, we examine the gut microbiota, which has the greatest bacterial abundance and diversity in the body (9). Immediately after birth, the gut microbiota starts to assemble alongside infant development, eventually forming a stable community. During this early stage, the developing microbiota is highly sensitive to external influences, with changes impacting long-term immune regulation, metabolism, and behavior (10–13). Therefore, infancy is a critical period of microbial and childhood development that has far-reaching impacts on later childhood health and disease.

**Figure 1:**
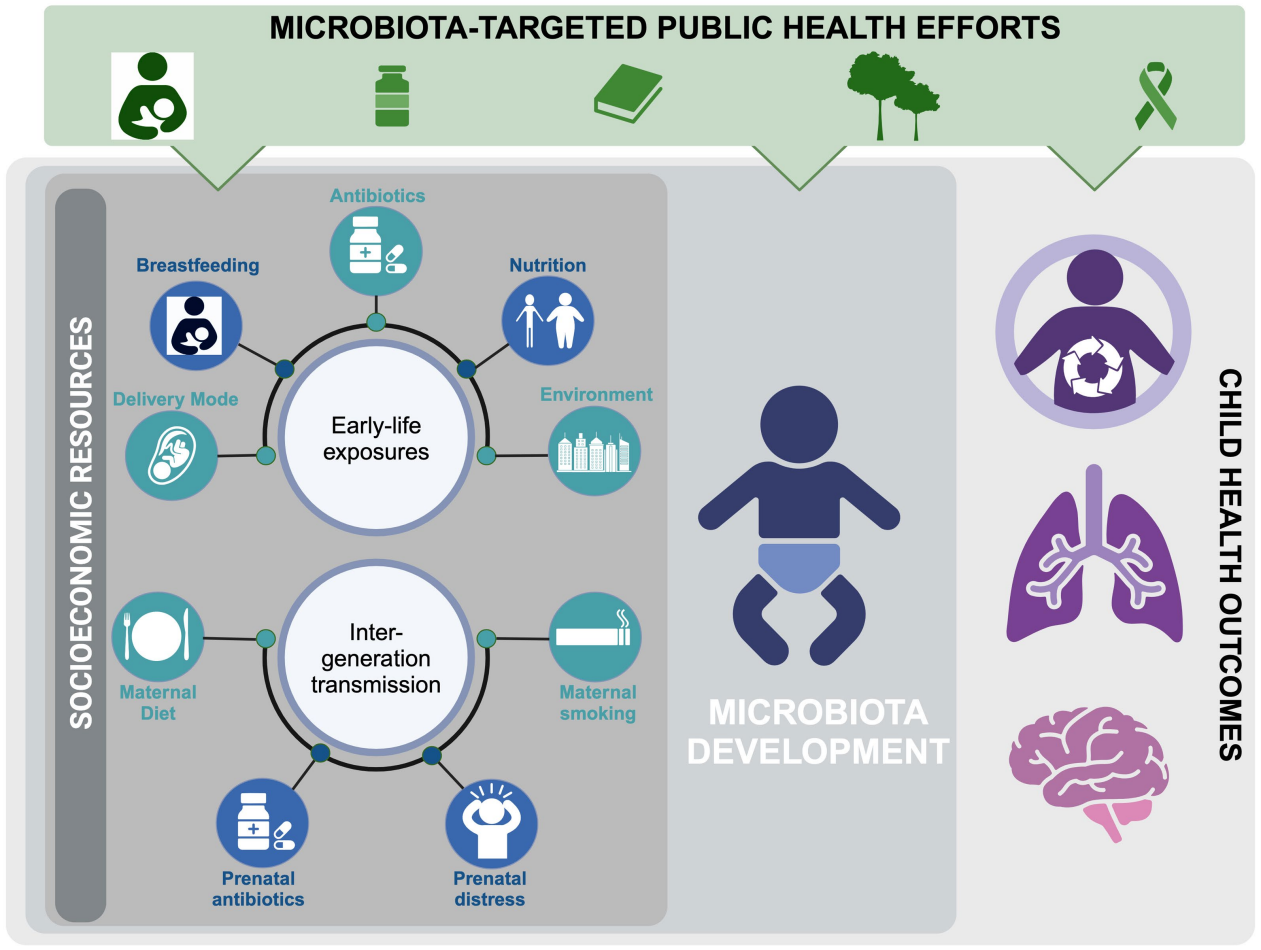
Factors influencing infant microbiome development, focusing on social inequality and potential public interventions to address disparities in child health.

Interest surrounding the impact of SES on the infant and childhood gut microbiota has grown in recent years, and many factors known to disrupt infant gut microbial communities (e.g., elevated cesarean-sections (C-sections), difficulty maintaining breastfeeding, reduced access to fresh foods, increased antibiotic exposure, and reduced proximity to greenspace) are also encountered by SES-disadvantaged populations and communities (14–19). As such, the role the microbiota plays in observed SES-mediated childhood health inequities is gradually gaining recognition. In this review, we will highlight findings linking SES-associated factors to the infant’s gut microbiota (Table 1). We will also explore accessible strategies for safeguarding and restoring the early-life microbiota in our society, with the goal of reducing health disparities for future generations (Figure 1).

**Table 1:** Effect of SES-associated factors on infant gut microbiota.

Early life and cross-generational exposures		Associated change in infant microbiota			Reference
Category	Factor	Diversity	Maturation	Composition	
Delivery mode	C-section	↓	↓	↓***Bacteroides*, *Bifidobacterium****, **Parabacteroides**,* ***Escherichia*** *^C^* ↑**Clostridiales**, **Enterobacteriaceae**^**C**^ **Fewer maternal microbes**^C^	(24, 25, 27, 35–39)
Breastfeeding	Human milk fed	↓	↓	↑***Bifidobacterium*, *Lactobacillus***^C^ ↓**Clostridiales**, **Enterobacteriaceae**, **Staphyloccoccaceae**^C^ **Cessation of BF drives the maturation of the infant gut, as marked by the phylum Firmicutes**^C^	(24, 27, 36, 37, 39, 52, 56, 57)
Antibiotics	Amoxicillin, penicillins, combination antibiotics, etc.	↓	↓	↓ ***Bifidobacterium***, **Clostridiales**, *Bacteroides fragilis*^C^ ↑ **γ-Proteobacteria**, Enterobacteriaceae, **ARGs**^C^	(35, 37, 38, 52, 59–61)
	Penicillin, vancomycin or combination	↓	n/a	↓Bacteroidetes^M^ ↑Firmicutes^M^	(62, 63)
	Streptomycin	NS	n/a	↑Bacteroidetes (Porphyromonadaceae and Bacteroidaceae)^M^ ↓Clostridiales^M^	
Malnutrition	Kwashiorkor/severe acute malnutrition	↓	↓	↓ ***Methanobrevibacter smithii***, ***Faecalibacterium prausnitzii***, ***Bifidobacterium longum*** and ***Lactobacillus*** mucosae^C^ ↑ ***Streptococcus gallolyticus***, **Proteobacteria** and Fusobacteria, **Bacteroidetes**, ***Desulfovibrio*** genus and Campylobacterales order^C^ ↑*B. wadsworthia* (related to ***Desulfovibrio***) and members of the order Clostridiales^C&M^	(78, 81–84)
	Malnourished diet (low protein & fat) vs. control	↑	n/a	↑Bacteroidetes, Proteobacteria^M^ ↓Lactobacillaceae^M^	(79)
	Low micronutrient diet (low vitamins, zinc and iron) vs. control	↑	n/a	↓Firmicutes and Erysipelotrichaceae^M^ ↑Proteobacteria and Enterobacteriaceae^M^	(80)
			Environment			Older sibling	↑	↑	1 m: ↑ ***Bifidobacterium**, Hungatella, Pediococcus* and ↓ *Clostridium*^C^ 6 m–1 y: ↓ *Escherichia/Shigella*, other Enterobacteriaceae, *Veillonella*, and ↑*Prevotella, Eisenbergiella*^C^	(27, 37, 56, 93)
Pets (dog, cat)	NS	↑		↑*Ruminococcus, Oscillospira*^C^ ↓Streptococcaceae^C^	(27, 94)
Farm or farm-like	NS	n/a		↑Bifidobacteriaceae *(B. infantis)*, Clostridiaceae, Aerococcaceae^C^	(95)
Air pollution (PM_10_, PM_2.5_, NO_2_)	NS	n/a		↑*Actinomyces* (Actinobacteria), *Clostridium*, *Enterococcus*, *Eubacterium* (Firmicutes), and *Haemophilus* (Proteobacteria) ↓*Alistipes*, *Phascolarctobacterium* (Proteobacteria)^C^	(96)
Maternal diet	Diet intake based on FFQ	n/a	n/a	aMED score: ↑Clostridiaceae spp. and ↓*Bacteroides uniformis, Escherichia coli, [Ruminococcus] gnavus*^C^ Fruit intake: ↑Clostridiaceae and ↓*Bifidobacterium*^C^ Fish intake: ↑*Streptococcus agalactiae* and ↓*Bacteroides uniformis*^c^ Dairy intake: ↑*Clostridium neonatale*, *C. butyricum*, *Staphylococcus* spp. and ↓*Lachnospiraceae* spp.^C^	(40)
	High fat intake vs. control (DSQ)	n/a	n/a	↑*Enterococcus* and ↓*Bacteroides*^C^	(104)
	Low-MAC	↓	n/a	↓Bacteroidales^M^	(105)
Prenatal antibiotics	Ampicillin, penicillin	↓	n/a	↓ ***Bifidobacterium***, ***Bacteroides***, *Blautia*, *Roseburia*, *Ruminococcus*, *Streptococcus* ↑**Proteobacteria**, *Escherichia*, *Oscillospora*, *Pseudobacter*, *Veillonella dispar*, ARGs^C^	(114, 115)
Prenatal distress	OASIS, PSS, PHQ	↓	n/a	↓*Bifidobacterium dentium*, *Bifidobacterium longum*, *Streptococcus salivarius*, *Lactobacillus rhamnosus*^C^	(120)
	EPDS, SCL, PRAQ	NS	n/a	↓*Akkermansia,* *Lactobacillus*^C^ ↑γ-Proteobacteria*^C^*	(121)
	Receive stress vs. control	↓	n/a	↓*Lactobacillus*, *Streptococcus*^M^	(122)
	Stress & delivery mode	NS	n/a	↓*E. coli, Streptococcus acidominimus, Streptococcus thoraltensis DSM12221, Lactobacillus murinus ASF361* and ↑Peptococcaceae^M^	(123)
Maternal smoke exposure	Prenatal and postnatal smoking	↑	n/a	1 m: ↑*Ruminococcus* and *Akkermansia*^C^ 3 m: ↑**Firmicutes** richness and diversity^C^ 6 m: ↑*Bacteroides* and *Staphylococcus*^C^	(128, 129)

This table describes how early-life exposures and intergenerational transmission factors affect the infant gut microbiome. Results with multiple pieces of evidence are highlighted in bold. NS, not significant; n/a, not available; C, clinical; M, mouse; ARG, antimicrobial resistance genes; aMED, alternative Mediterranean diet; FFQ, food frequency questionnaire; DSQ, Dietary Screener Questionnaire; MAC, microbiota-accessible carbohydrates (dietary fiber); OASIS, Overall Anxiety Severity and Impairment Scale; PHQ, Patient Health Questionnaire; PSS, Perceived Stress Scale; EPDS, Edinburgh Postnatal Depression Scale; SCL-90, Symptom Checklist-90; PRAQ-R, The 10-item Pregnancy-Related Anxiety Questionnaire–Revised.

## Infant microbiome development

Before exploring the links between social inequity and the infant microbiome, it is important to discuss our current understanding of microbiota development. The primary seeding point for the gut microbiota is at birth, which is significantly influenced by the delivery mode, maternal microbiota, and maternally-derived metabolites and immune components (20–25). Initial colonization is primarily dominated by aerobes and facultative anaerobes, such as *Staphylococcus* and γ-Proteobacteria, which gradually reduce oxygen to support anaerobic colonizers, such as *Bifidobacterium*, *Bacteroides*, and multiple Clostridia genera (26). Within the first few months of life, species diversity is limited and composition is primarily dictated by type of milk diet (27). Later, as infants experience more varied environments and diets, their microbiota diversity increases. As such, prior to reaching a stable community, the microbiota matures in the first 1–3 years through patterned temporal shifts in species abundances that can be commonly detected across studies (11, 28, 29). Supporting the importance of this maturation process, microbiota disruptions during infancy—but not later—are broadly associated with a higher likelihood of allergic disease, obesity, and behavioral disorders (11, 12, 29, 30). Therefore, prioritizing normative microbiota maturation by limiting disruptions or implementing restorative interventions during this sensitive window can yield lifelong health benefits.

## Potential pathways for social inequity to influence the infant microbiome

### Effects of early-life exposures

#### Delivery mode

The mode of delivery is a primary driver of the initial compositional variance in the infant gut microbiota. However, multiple independent studies within developed nations have shown that women from lower SE backgrounds have a higher likelihood of undergoing C-section deliveries (31–34). Compared to infants born vaginally, infants born via C-sections harbor fewer maternal bacterial strains, display reduced diversity, and tend to have fewer *Bacteroides* and *Bifidobacterium*; and these alterations are independent of additional exposures, such as antibiotic use during delivery and a reduced ability to initiate breastfeeding (24, 25, 27, 35–40). However, the impact of this on later health is still unclear. Although a C-section delivery within some studies has been implicated in an elevated risk of childhood obesity, asthma and immune disorders (41–43), others have not observed any correlation (44, 45). This likely underscores a compounding effect of risk factors and implies that the negative outcomes associated with C-section deliveries could be outweighed by subsequent early-life experiences.

#### Breastfeeding

Immediately after birth, the diverse array of human milk components is crucial for shaping the infant’s gut microbiota and supporting infant development (36, 39, 46, 47). These include secretory IgA (sIgA) (48), the milk microbiota, which serves as a significant seeding source (25, 49), and metabolites such as human milk oligosaccharides (HMOs), which serves as the third most abundant milk metabolite yet primarily serve to nourish ‘infant-type’ bacteria (50). As a constant force on the infant microbiome, breastfeeding can mitigate disruptions caused by both C-sections and antibiotic exposure (51–53). However, lower-income families often report difficulty initiating breastfeeding and shorter overall breastfeeding durations (54, 55). This has the potential to profoundly impact the infant microbiota, as formula-fed babies experience premature microbiota maturation, reduced abundance of *Bifidobacterium*, and more opportunistic pathogens compared to their breastfed counterparts (24, 27, 36, 37, 39, 52, 56, 57). These differences are potentially associated with child health consequences, as evidenced by a large, nationally representative longitudinal study in the United States (*n* = 8,030 participants), which identified infant feeding practices as a significant mediator between SES and early childhood obesity (58). Thus, greater support is needed for mothers and families who would otherwise breastfeed their infants, but are unable to do so.

#### Antibiotics

Antibiotics are a potent medical intervention that reduces or depletes pathogenic microbes and has considerably reduced human mortality since their discovery. However, antibiotics, especially broad-spectrum ones, commonly target both pathogenic and beneficial commensal bacteria indiscriminately; and extensive evidence exists for their off-target disruption of infant gut microbiota homeostasis. The detrimental effects include a reduction in species diversity, an enrichment of antimicrobial resistance genes (ARGs), the loss of beneficial bystanders such as *Bifidobacterium* and Clostridia members, and elevated γ-Proteobacteria during recovery (35, 37, 38, 52, 59–61). In addition, a number of animal studies provide evidence of the causal impact of early-life antibiotic-induced microbiota disruptions on long-term host health (62, 63). Infancy is particularly sensitive to the disruptions of early-life antibiotic exposure, which is widely associated with subsequent risk of inflammatory bowel disease, atopy, asthma, and obesity (61, 64–68). Despite being intrinsically linked to medical care access, lower SES in wealthy countries is paradoxically associated with higher early-life antibiotic exposure (69–71). Indeed, a large US study found that children from lower SES families, particularly in high-poverty areas, received more antibiotics in the first month of life despite receiving fewer antibiotic prescriptions over their lifetime (72). More alarming trends are seen from children in low- and middle-income countries, who are prescribed an average of 25 antibiotic prescriptions during their first 5 years of life. This is a remarkable amount considering that two antibiotic prescriptions per year are considered excessive in many high-income countries (73). Antibiotic stewardship initiatives, which primarily aim at reducing the spread of antimicrobial resistance, may therefore possess the additional benefit of preserving the healthy infant microbiota (60, 71, 74, 75).

#### Malnutrition

Malnutrition can be characterized by the over- or under-bioavailability of both macro- and micronutrients, and is commonly observed in low SES and minoritized populations (2, 76, 77). Due to its capacity to regulate nutritional harvest, the gut microbiota is an important nexus between diet and health outcomes (30). This is evidenced by a longitudinal study of Malawian twins, which found that poorly matured gut microbiota was associated with malnutrition and the causal relationship between microbiota and malnutrition is supported by multiple independent mouse studies (78–80). Malnutrition is associated with reduced obligate anaerobic species and an increase in potentially pathogenic microbes in the infant gut microbiome (78, 81–84). Furthermore, this phenomenon is widespread, as multiple studies in low-SES countries have consistently demonstrated decreased bacterial diversity in malnourished children, reduced beneficial microbes and increased pathogen enrichment (82, 84, 85). Recent randomized clinical trials have established that microbiota-directed foods, but not caloric intake alone, successfully support growth recovery in malnourished children (86, 87). These findings confirm the microbiota’s role in malnutrition and emphasize the need for microbiota-informed interventions in its management.

#### Environment

The environment to which humans are exposed throughout their lives is a complex and important determinant of microbiota composition, with broad implications for NCDs (88–92). The infant microbiota is particularly reflective of its surroundings, with exposures from air pollution, older siblings, pets, and farms all linked to differences in composition (27, 37, 56, 93–96). Furthermore, these have enduring impacts on both the microbiota community and our health. For instance, a comprehensive Dutch study found that childhood living environments were significantly associated with adult microbiota composition despite only a weak association between childhood and adult urbanicity (97). Similarly, research has shown that children who lived on farms or near farm-like environments during the perinatal period had a lower risk of asthma later in life (98, 99). Given that an increase in SES in urban environments is positively linked to surrounding greenspace and biodiversity, and that SES-disadvantaged neighborhoods exhibit measurably reduced microbiota diversity (92), efforts to ‘rewild’ our cities and expanding public access to greenspaces could be incredibly effective ways to facilitate these vital health-promoting microbial exposures.

### Intergenerational transmission

#### Maternal diet

The quality of the maternal diet is heavily influenced by the resources afforded by SES (100–102). Not only do mothers’ dietary patterns influence their own microbiota, they are also determinants of health outcomes during and after pregnancy (102, 103). As such, maternal diet can significantly influence the infant microbiota via impacting breastfeeding outcomes, altering breastmilk components, or influencing maternal microbes that seed the infant (40, 104). Importantly, these effects can persist or even compound over generations (105). The exact impacts that maternal diet has on health outcomes of offspring are best measured in well controlled animal models. For instance, after researchers in China found that maternal obesity affected child neurodevelopment, they were able to mirror the phenomenon in mice by maternal fecal microbial transplantation (FMT) and reverse the phenotype by feeding the dams high-fiber diets (106). Similarly, a maternal low-fiber diet in mice predisposed offspring to severe lower respiratory tract infections and asthma, and milk microbes from dams fed a high-fiber diet were able to rescue this effect (107). Therefore, prioritizing access to nutritiously complete maternal diets may mitigate detrimental cross-generational impacts of low SES on infant and child health.

#### Prenatal antibiotics

The relationship between SES and prenatal antibiotic use remains unclear, with results being largely dependent on the specific SES measures, study cohort demographics, and economic status of the origin country (108–110). However, we mention it because, despite birth being a crucial seeding point for the microbiota, the rates of antibiotic prescription during pregnancy and birth are markedly high. Indeed, in Western countries, between 30 and 40% of women receive antibiotics either prenatally or during birth, with the majority prescribed for prophylactic reasons (111–113). This has real consequences on the neonatal microbiota. Infants with mothers exposed to antibiotics during pregnancy are reported to have reduced microbial diversity, less abundant Bacteriodetes and Bifidobacteria, and expanded γ-Proteobacteria (114, 115). Importantly, while the risks associated with antibiotic disruptions can be mitigated with breastfeeding, this may be less available to mothers of lower SES, particularly in countries with reduced access to paid maternity leave (52, 116).

#### Prenatal distress

The strong link between lower SES and prenatal distress is associated with several adverse health outcomes in offspring, such as preterm birth, low birth weight, and negative neurodevelopmental outcomes (117–119). Various changes have been reported between infant microbiota and different indicators of prenatal stress (e.g., subjective distress, cortisol level, precarity, prenatal anxiety, depression, and perceived stress), the most consistent being enrichment of γ-Proteobacteria and reduction in *Bifidobacterium*. Although reductions in species diversity have been reported, they have not been as consistent and appear to vary depending on the measure and timing of distress, as well as the age of the offspring (120, 121). These effects may be partially explained by altering gut microbial composition during pregnancy and transmission of disrupted vaginal microbiome at birth based on mouse models (122, 123).

#### Maternal smoke exposure

Low SES is a risk factor for smoking, and maternal smoking during pregnancy is associated with increased incidence of premature birth, childhood obesity, developmental delays, respiratory disease, and long-term morbidity among offspring (124–127). Evidence connecting maternal smoke exposure to the infant gut microbiota is limited, but consistent. A review of three studies found that infants exposed to maternal smoke had an increased Firmicutes richness, which was associated with an elevated risk of childhood overweight and obesity (128). Similar findings were independently observed in a recent study using the cohort data from the Canadian Healthy Infant Longitudinal Development (CHILD) study, Canada, which revealed that maternal smoking during pregnancy increases the risk of being overweight at 1 and 3 years, and this was mediated by an increase in Firmicutes diversity (129). Interestingly, although smoking cessation during pregnancy does not reduce the risk of offspring being overweight, exclusive breastfeeding does.

## Utilizing the gut microbiome to tackle disparities in health

Given the impact SES-linked factors have on the infant microbiota and long-term health, prioritizing healthy microbiota development during infancy may alleviate health inequities for future generations. Considerable attention has been given in other reviews to highlight specific avenues that reduce disruptions, reinforce health-promoting microbial interactions, and replenish important species when they are lost (10, 130–132). We will highlight methods that can be broadly integrated into public health efforts to inclusively bolster infant microbiota development throughout our society.

### Reduce: promote antibiotic stewardship

An increasing number of antibiotic stewardship programs (ASPs) have been successfully implemented worldwide, leading to reduced antibiotic use, improved clinical and microbiological outcomes and economic benefits, indicating the effectiveness of this public health intervention (133–136). While the public focus on antibiotic stewardship has been aimed at limiting antimicrobial resistance, an unintended benefit may be protecting vital microbial species within the infant microbiota, which reduce instances of childhood NCDs. Supporting this, our own findings linked recent declines in antibiotic prescriptions within British Columbia, Canada to lower rates of pediatric asthma, with the infant microbiota demonstrated to be the likely mediator of this relationship (60). As additional public health ASPs are launched, it will be interesting to observe if these trends are recapitulated across communities.

### Reinforce: support breastfeeding and access to donor milk

Despite the clear benefits of breastfeeding and recommendations to initiate within the first hour of life and exclusively breastfeed for the first 6 months, SES-disadvantaged families often have lower breastfeeding initiation and duration rates (137). This can be attributed to barriers such as a lack of breastfeeding educational resources, an absence of breastfeeding experience within previous generations, and employment and financial limitations (132, 138, 139). Some of these can be addressed at the individual or community level, as evidenced by randomized controlled trials that have successfully increased breastfeeding motivation and success (140, 141). However, societal-level policy and fiscal support, such as generous, universally paid parental leave, are needed to decrease the chasm between low- and high-SES families (132, 142). Furthermore, when breastfeeding is just not an option, regardless of support, standard care that includes access to donor breast milk or prioritized research into formulas that mimic human milk is needed to support the infant microbiota (143, 144). All of these in parallel are necessary to improve long-lasting cross-generational health outcomes and reduce health disparity.

### Replenish: restore and boost exposure to missing microbes

Many researchers now believe that our society’s intergenerational loss of microbiota biodiversity has paralleled the rise in NCDs (145, 146). This indicates that reinforcements, such as breastfeeding, alone may be insufficient and will need to be complemented by reintroducing key species to infants. Despite the efficacy of full microbiota replacement through FMT for severe diseases such as *Clostridioides difficile* in older populations, it is unlikely to serve as a preventative measure in infants (147). Even randomized controlled trials that effectively swabbed C-section born neonates with maternal vaginal microbes faced significant opposition from the medical community (148, 149). More feasible are live biotherapeutic products (LBPs) that contain rigorously controlled and tested microbial species to replenish key missing microbes. One excellent example is *B. infantis*, a crucial HMO-utilizing microbe that has declined in North America (95, 150–152). To combat this, community-based stool testing during checkups could identify infants who would benefit from a *B. infantis* LBP, which has been shown to successfully colonize infants to reduce gut dysbiosis and shape immune development (151, 153–155). Beyond *B. infantis*, *Bifidobacterium* and *Lactobacillus* contain a number of secure and effective species that can colonize both breastmilk and neonate gut microbiota when administered to mothers during pregnancy or lactation (156, 157). However, studies supporting their utility for long-term colonization and health benefits are still lacking (158, 159). In addition to targeted microbial restoration, a broader “rewilding” of urban environments could provide widespread and lasting benefits to counter biodiversity loss. Indeed, the soil microbiota found in revegetated urban spaces mimics that of natural vegetation, supporting the feasibility of reintroducing wild microbes into urban environments. Naturalized greenspaces can boost microbial biodiversity in young children, with measurable impacts benefitting tolerant immune responses (160, 161). In addition, there is more work to be done in public health policy to mitigate SES-associated microbiota and health inequity, such as making healthcare more affordable, providing education to the general public (i.e., hygiene hypothesis), and increasing access to fresh foods. Collectively, public health initiatives may be able to equitably restore necessary early-life microbiota interactions at the community level, thereby reducing or preventing microbiota-associated SES disparities and ensuring equitable health outcomes.

## Conclusion

Over the past several decades, growing evidence has demonstrated the impact of early-life and cross-generational factors on infant microbiota development. These factors include delivery mode, breastfeeding, maternal stress, diet, and urbanization, all of which are commonly associated with SES. Since microbiota composition is modifiable and associated with infant immune development and long-term health, it represents an important opportunity to mitigate the impact of SES inequity on health disparity. In this context, broadly integrated biomedical and policy interventions aimed at reducing, reinforcing, and replenishing the disrupted microbiota should be adopted and provided at equalizing access. In addition, further studies aimed at understanding SES-associated microbiota diversity, composition, and functions in large-scale, diverse, and longitudinal settings are needed to better understand the variations in gut microbiota across diverse geographies, ethnicities, lifestyles, and ages.
